# Clinical usefulness of NT-proBNP as a prognostic factor for septic shock patients presenting to the emergency department

**DOI:** 10.1038/s41598-024-61888-5

**Published:** 2024-05-14

**Authors:** Yunhyung Choi, Jae Hee Lee

**Affiliations:** 1 Chung-Ang University Gwangmyeong Hospital, Deokan-ro 110, Gwangmyeong-si, Gyeonggi-do 14353 Republic of Korea; 2 Department of Emergency Medicine, College of Medicine, Ewha Womans University Mokdong Hospital, Ewha Womans University, 1071 Anyangcheon-ro, Yangcheon-gu, Seoul, 07985 Republic of Korea

**Keywords:** Sepsis, Septic shock, Natriuretic peptides, Brain natriuretic peptides, Biomarkers, Diseases

## Abstract

Plasma N-terminal prohormone of brain natriuretic peptide (NT-proBNP) level is primarily used as a biomarker for left ventricular (LV) dysfunction. It is influenced by various conditions, such as myocardial strain and situations affecting the clearance of NT-proBNP, including sepsis and shock. In this study, we investigated the appropriateness of NT-proBNP as a prognostic factor for septic shock. Patients with septic shock who visited the emergency department of the Ewha Womans’ University Mokdong Hospital between January 1, 2018, and December 31, 2020, were classified into the survival group (those who survived in the hospital and were discharged) and the death group (those who died in the hospital). The effectiveness of NT-proBNP, lactate, and blood urea nitrogen as predictive factors of in-hospital mortality was evaluated using the area under the receiver operating characteristic (AUROC) curve. The AUROC curve was 0.678 and 0.648 for lactate and NT-proBNP, respectively, with lactate showing the highest value. However, there was no significant difference between lactate and NT-proBNP levels in the comparison of their AUROC curve (*p* = 0.6278). NT-proBNP could be a useful predictor of in-hospital mortality in patients with septic shock who present to the emergency department.

## Introduction

Sepsis is a life-threatening organ dysfunction resulting from a dysregulated host response to infection. Septic shock is a progression of sepsis in which the underlying circulatory and cellular metabolic abnormalities are severe enough to significantly increase mortality^1^. Sepsis is characterized by rapid progression that can lead to multiple organ dysfunction syndrome and high mortality rates^2–4^. Sepsis continues to be the primary contributor to hospital fatalities, with mortality rates ranging from approximately 15–20%, as reported in previous clinical trials^5^. Notably, the mortality rate escalates to about 50% in the critically ill subset of individuals experiencing septic shock^1,6,7^. The medical treatment cost for patients with sepsis is increasing continuously. In the United States, the total cost of hospitalizations and skilled nursing facility admissions for Medicare A/B/C patients increased from $27.7 billion in 2012 to $41.5 billion in 2018^8^. In South Korea, the standardized medical costs for sepsis increased by 75.5% from 2005 to 2012^9^.

Despite numerous discussions on the diagnosis and management of sepsis, it remains challenging owing to its various etiologies and clinical presentations^1,10–18^. Sepsis exhibits evolving characteristics over time, and sepsis-induced organ dysfunction may be occult. Contrastingly, septic shock sends an immediate danger signal to clinicians owing to a rapid decrease in blood pressure, which is subsequently diagnosed through interventions such as administering vasopressors or fluid resuscitation and lactate tests^1^.

To date, little distinction has been made between sepsis and septic shock, and many studies have focused on biomarkers^19,20^. Sepsis is difficult to detect, requiring various scoring systems^21^ and continuous monitoring. Septic shock can be immediately recognized through the patient’s clinical status and has a higher mortality rate than sepsis. Emergency medicine doctors often encounter patients with unstable vital signs due to septic shock. Identifying predictive factors for in-hospital mortality in septic shock will ultimately help increase patient survival rates through the efficient operation of limited emergency department (ED) staff and medical resources. In patients with unstable vital signs, N-terminal prohormone of brain natriuretic peptide (NT-proBNP) levels are measured to detect left ventricular dysfunction. NT-proBNP levels can also be used as a predictor of sepsis^22^. In this study, we conducted an analysis to investigate the suitability of NT-proBNP as a predictive factor in patients with septic shock who present to the emergency department.

## Methods

### Study design and data collection

This study was a retrospective data analysis of patients visiting the ED of Ewha Womans University Mokdong Hospital between January 1, 2018 and December 31, 2020. The number of annual visits to the ED was approximately 40,000. In this study, we selected patients who received intravenous antibiotics in the ED during the study period and chose those with quick Sepsis-related Organ Failure Assessment (qSOFA, i.e., alteration in mental status, systolic blood pressure (SBP) ≤ 100 mmHg, or respiratory rate (RR) ≥ 22/min) scores ≥ 2 as patients with sepsis^1,21^. Seymour et al. reported that qSOFA and SOFA have similar predictive validity in the outside ICU setting, and based on its clinical utility, we adopted qSOFA as a diagnostic tool^21^. Among them, patients who received vasopressors to maintain mean arterial pressure > 65 mmHg after an initial fluid bolus of 20 ~ 30 ml/kg/hr were defined as the final study population for septic shock. Although lactate levels after fluid administration would also ideally be included in the diagnostic criteria, we did not include patients with serum lactate levels > 2 mmol/L (18 mg/dL) in the selection of patients with septic shock because lactate levels were measured as an initial blood test when sepsis was suspected, but this is not routinely performed after fluid administration.

Pediatric patients (aged < 18 years) were excluded from the study. Patients with out-of-hospital cardiac arrest, trauma, or non-infectious diseases were excluded, as were those for whom NT-proBNP levels in the blood were not measured. The survival and death groups were determined according to the results of discharge from the ED or hospital.

Data including age, sex, medical history, chief complaint, mental state, and vital signs were recorded immediately after the patients arrived at the ED. Blood examinations, including complete blood count, blood urea nitrogen (BUN), creatinine, C-reactive protein (CRP), procalcitonin, NT-proBNP, lactate, ketone, arterial pH, and blood culture, were performed immediately after each patient arrives at the ED. All data were recorded using an electronic medical recording system. Two board-certified emergency physicians selected and analyzed the data.

### Statistical analysis

The Mann–Whitney U test was used for the analysis of continuous variables, while the chi-squared test or Fisher’s exact test was used for categorical variables, depending on appropriateness. Quantitative data are presented as medians with interquartile ranges, whereas categorical data are expressed as numbers and percentages. Statistical significance was set at a two-tailed *p-*value < 0.05. Analysis was conducted using the Statistical Package for the Social Science (SPSS) version 26.

To assess predictive accuracy, receiver operating characteristic (ROC) curve analysis was performed for WBC, neutrophil, BUN, creatinine, CRP, lactate, procalcitonin, NT-proBNP, and arterial pH. MedCalc statistical software version 19.4.1 was employed for the ROC curve analysis.

The method of DeLong et al.^23^ was used to calculate the standard error of the area under the curve (AUC) and the difference between the two AUCs. The predictive accuracy for in-hospital mortality was compared among early blood test measurements using area under the ROC curve (AUROC) and 95% confidence interval (CI).

The optimal cutoff points for each blood test were determined using the Youden Index of ROC curves. Sensitivity, specificity, positive likelihood ratio (+ LR), and negative likelihood ratio (-LR). CI was used to estimate the prognostic accuracy of each criterion for the proposed cutoff points.

### Ethics statement

This study was approved by the Institutional Review Board (IRB) of the Ewha Womans University Mokdong Hospital (IRB No. 2023–04-008). All methods were carried out in accordance with relevant guidelines and regulations. The need for informed consent was waived by the IRB of Ewha Womans University Mokdong Hospital (IRB No. 2023–04-008) because of the retrospective nature of the study, and patient information was anonymized before analysis to ensure confidentiality.

## Results

### Patient baseline

**Figure 1:**
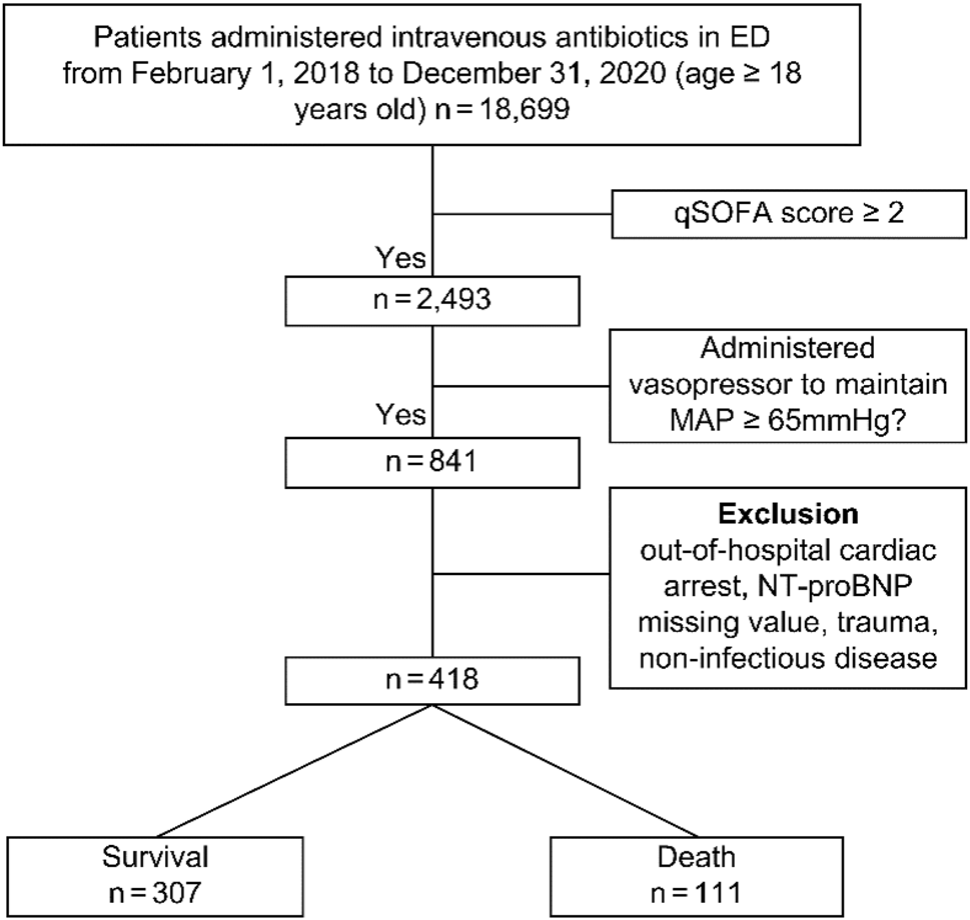
Flow chart of the study participants. *ED* Emergency department, *qSOFA* quick Sepsis-related Organ Failure Assessment, *MAP* mean arterial pressure, *NT-proBNP* N-terminal prohormone of brain natriuretic peptide.

The participants were 418 patients with septic shock (Fig. 1). During the study period, 307 patients survived and were discharged (survival group), and 111 patients died (death group) (Table 1). The median age of the survival group was 76 years old (IQR: 64.00–82.00), and that of the death group was 79 years old (IQR: 70.00–84.00, *p* < 0.05). The study sample comprised 241 males (57.7%) and 177 females (42.3%). In both groups, most patients with septic shock were hypertensive (143 and 50 patients, respectively), and the only significant difference between the groups was the incidence of stroke (*p* < 0.05). The most common symptom reported in both groups was dyspnea (survival vs. death; n = 85, 27.7% vs. n = 42, 37.8%). The second most frequently reported symptom differed between the two groups: it was fever in the survival group (n = 77, 25.1%) but altered mental status in the death group (n = 28, 25.2%, *p* < 0.05). At the time of arrival in the ED, the level of consciousness was predominantly alert in the survival group (n = 160, 52.1%), whereas the death group had a higher proportion of patients with pain-responsive consciousness (n = 43, 38.7%; *p* < 0.05).

**Table 1:** Baseline data of patients with septic shock presenting to the emergency department.

	Survival	Death	Total	*p*-value
	n	n	n	
No. of patients	307 (73.4)	111 (26.6)	418 (100)	
Age (yrs)	76.00 (64.00–82.00)	79.00 (70.00–84.00)	77.00 (65.00–82.00)	0.006
Sex				0.373
Male	173 (56.4)	68 (61.3)	241 (57.7)	
Female	134 (43.6)	43 (38.7)	177 (42.3)	
Underlying disease				
DM	107 (34.9)	38 (34.2)	145 (34.7)	0.908
HTN	143 (46.6)	50 (45.0)	193 (46.2)	0.825
Stroke	86 (28.0)	20 (18.0)	106 (25.4)	0.042
Heart disease	53 (17.3)	15 (13.5)	68 (16.3)	0.374
Asthma	14 (4.6)	6 (5.4)	20 (4.8)	0.796
COPD	17 (5.5)	7 (6.3)	24 (5.7)	0.812
Renal failure (CKD)	24 (7.8)	12 (10.8)	36 (8.6)	0.430
Others	156 (50.8)	59 (53.2)	215 (51.4)	0.740
No basic disease	16 (5.2)	10 (9.0)	26 (6.2)	0.171
Chief complaint				0.003
Abdominal pain	20 (6.5)	7 (6.3)	27 (6.5)	
Altered mentality	42 (13.7)	28 (25.2)	70 (16.7)	
Dyspnea	85 (27.7)	42 (37.8)	127 (30.4)	
Fever	77 (25.1)	11 (9.9)	88 (21.1)	
General weakness	29 (9.4)	8 (7.2)	37 (8.9)	
Hypotension	23 (7.5)	6 (5.4)	29 (6.9)	
Others	31 (10.1)	9 (8.1)	40 (9.6)	
Mental state				0.006
Alert	160 (52.1)	40 (36.0)	200 (47.8)	
Verbal responsive	62 (20.2)	28 (25.2)	90 (21.5)	
Pain responsive	79 (25.7)	43 (38.7)	122 (29.2)	
Unresponsiveness	6 (2.0)	0 (0.0)	6 (1.4)	
Vital sign				
SBP (mmHg)	85.00 (73.50–97.00)	88.50 (68.50–103.75)	85.00 (73.00–98.00)	0.473
DBP (mmHg)	50.00 (41.00–61.00)	51.50 (42.00–61.00)	51.00 (41.50–61.00)	0.803
HR (/min)	105.00 (90.00–122.25)	107.00 (84.00–124.00)	105.00 (89.00–123.00)	0.864
RR (/min)	22.00 (20.00–25.00)	23.50 (20.00–27.00)	22.00 (20.00–26.00)	0.070
BT (°C)	37.30 (36.40–38.30)	36.80 (36.00–37.80)	37.20 (36.20–38.20)	0.010

Quantitative data are expressed as mean (± standard deviation) or median (interquartile range), and categorical data are presented as numbers (percentage). Student’s *t* test or Mann–Whitney U test was used for continuous variable analysis, while the chi-squared test or Fisher’s exact test was used for categorical variable analysis, as appropriate.

*DM* diabetes mellitus; *HTN* hypertension; *COPD* chronic obstructive pulmonary disease; *CKD* chronic kidney disease; *SBP* systolic blood pressure; *DBP* diastolic blood pressure; *HR* heart rate; *RR* respiratory rate; *BT* body temperature.

The two groups included patients with septic shock, and their overall vital signs were as follows: SBP: 85.00 mmHg (IQR: 73–98.00), diastolic blood pressure: 51.00 mmHg (IQR: 41.50–61.00), heart rate: 105.00/min (IQR: 89.00–123.00), RR: 22.00 (IQR: 20.00–26.00), and body temperature: 37.2 °C (IQR: 36.20–38.20). There was a significant difference in body temperature between the groups (*p* < 0.05).

### Comparison of laboratory test and management

Among the various blood tests performed (Table 2), only BUN, creatinine, CRP, procalcitonin, NT-proBNP, lactate, and arterial pH showed significant differences between the groups (*p* < 0.05).

**Table 2:** Comparison of laboratory test and management between survival and death group of patients with septic shock presenting to the emergency department.

	Survival	Death	Total	*p*-value
	n	n	n	
Laboratory test				
WBC (× 10^3^/ul)	12.69 (8.40–18.52)	10.96 (6.30–17.67)	12.48 (7.46–18.31)	0.067
Neutrophil (%)	87.20 (80.50–91.80)	87.50 (77.30–92.30)	87.30 (80.00–92.00)	0.868
BUN (mg/dL)	30.50 (21.00–46.25)	40.00 (28.00–59.00)	33.00 (23.00–52.00)	0.000
Creatinine (mg/dL)	1.50 (1.02–2.34)	1.82 (1.20–3.06)	1.59 (1.05–2.49)	0.006
CRP (mg/dL)	11.33 (5.09–19.27)	15.07 (9.12–25.62)	12.58 (6.24–20.23)	0.001
Procalcitonin (ng/mL)	3.39 (0.89–19.72)	6.47 (1.49–37.35)	4.39 (1.02–23.60)	0.027
NT-proBNP (pg/mL)	2,158.00 (668.00–6,075.00)	4,133.00 (1743.00–11,693.00)	2,726.50 (883.33–7,202.25)	0.000
Lactate (mg/dL)	27.00 (16.00–46.00)	45.00 (26.50–71.00)	30.00 (18.00–52.00)	0.000
Ketone (umol/L)	257.60 (99.30–669.20)	346.30 (124.80–853.60)	275.75 (105.15–715.90)	0.231
pH, arterial	7.41 (7.33–7.47)	7.36 (7.24–7.43)	7.39 (7.31–7.46)	0.000
Source of infection				
Respiratory	180 (58.6)	79 (71.2)	259 (62.0)	0.022
Intra-abdominal	85 (27.7)	23 (20.7)	108 (25.8)	0.165
Genitourinary	95 (30.9)	14 (12.6)	109 (26.1)	0.000
Skin, soft tissue	15 (4.9)	7 (6.3)	22 (5.3)	0.621
Others	30 (9.8)	19 (17.1)	49 (11.7)	0.057
Antibiotics				0.028
Meropenem	94 (30.6)	25 (22.5)	119 (28.5)	
Pip/tazo	75 (24.4)	32 (28.8)	107 (25.6)	
Ceftriaxone	33 (10.7)	6 (5.4)	39 (9.3)	
Meropenem + vancomycin	21 (6.8)	18 (16.2)	39 (9.3)	
Pip/tazo + meropenem	15 (4.9)	5 (4.5)	20 (4.8)	
Ceftriaxone + meropenem	16 (5.2)	3 (2.7)	19 (4.5)	
Cefepime	7 (2.3)	8 (7.2)	15 (3.6)	
Ceftriaxone + pip/tazo	9 (2.9)	1 (0.9)	10 (2.4)	
Pip/tazo + meropenem + vancomycin	4 (1.3)	3 (2.7)	7 (1.7)	
Meropenem + teicoplanin	4 (1.3)	3 (2.7)	7 (1.7)	
Others	29 (9.4)	7 (6.3)	36 (8.6)	
Inotropes or vasopressor				0.000
NE	260 (84.7)	49 (44.1)	309 (73.9)	
NE + VA	20 (6.5)	18 (16.2)	38 (9.1)	
NE + VA + EP + DA	2 (0.7)	11 (9.9)	13 (3.1)	
NE + VA + EP	5 (1.6)	5 (4.5)	10 (2.4)	
NE + EP	3 (1.0)	6 (5.4)	9 (2.2)	
Others	17 (5.5)	22 (19.8)	39 (9.3)	
Duration of administration of vasopressors (days)	2.00 (1.00–4.00)	3.00 (2.00–7.00)	2.00 (1.00–4.00)	0.000
Applying mechanical ventilation	80 (26.1)	67 (60.4)	147 (35.2)	0.000
Applying continuous renal replacement	20 (6.5)	24 (21.6)	44 (10.5)	0.000
Micro-organism in blood culture				
Gram ( −) rod	83 (27.0)	24 (21.6)	107 (25.6)	0.310
Gram ( +) cocci	50 (16.3)	21 (18.9)	71 (17.0)	0.556
No growth	175 (57.0)	65 (58.6)	240 (57.4)	0.823
Others	7 (2.3)	3 (2.7)	10 (2.4)	0.729
ED length of stay in hours	6.13 (4.85–8.32)	6.12 (4.47–9.05)	6.13 (4.75–8.68)	0.752
Hospital length of stay in days	14.18 (6.91–25.89)	3.24 (0.82–13.73)	14.79 (7.60–26.73)	0.000

Quantitative data are expressed as mean (± standard deviation) or median (interquartile range), and categorical data are presented as numbers (percentage). Student’s *t* test or Mann–Whitney U test was used for continuous variable analysis, while the chi-squared test or Fisher’s exact test was used for categorical variable analysis, as appropriate.

*Pip/tazo* Piperacillin/tazobactam, *NE* norepinephrine; *VA* vasopressin; *DA* dopamine; *EP* epinephrine.

Respiratory tract infection was the most common infection in both groups (survival vs. death; 58.6% vs. 71.2%), followed by genitourinary tract infection (n = 95, 30.9%) in the survival group and intra-abdominal infection (n = 23, 20.7%) in the death group. The most common antibiotics used in all patients with septic shock were meropenem (n = 119, 28.5%) and piperacillin/tazobactam (n = 107, 25.6%). The two groups predominantly used norepinephrine alone (survival vs. death; n = 260, 84.7% vs. n = 49, 44.1%), and the duration of inotrope or vasopressor use was 2.00 days (IQR: 1.00–4.00) in the survival group and 3.00 days (IQR 2.00–7.00) in the death group (*p* < 0.05).

Patients who received mechanical ventilation had a rate of 26.1% (n = 80) in the survival group compared with 60.4% (n = 67, *p* < 0.05) in the death group. Among the patients who received continuous renal replacement therapy, the survival group had a rate of 6.5% (n = 20), whereas the death group had a rate of 21.6% (n = 24, *p* < 0.05). The blood culture test did not detect any bacterial growth in 57.0% of the patients in the survival group and in 58.6% in the death group. The length of stay in the ED was 6.13 h (IQR: 4.75–8.68, *p* = 0.752) overall, and the total length of hospital stay was 14.18 days (IQR: 6.91–25.89) for the survival group and 3.24 days (IQR: 0.82–13.73, *p* < 0.05) for the death group.

### NT-proBNP as a predictive factor

**Figure 2:**
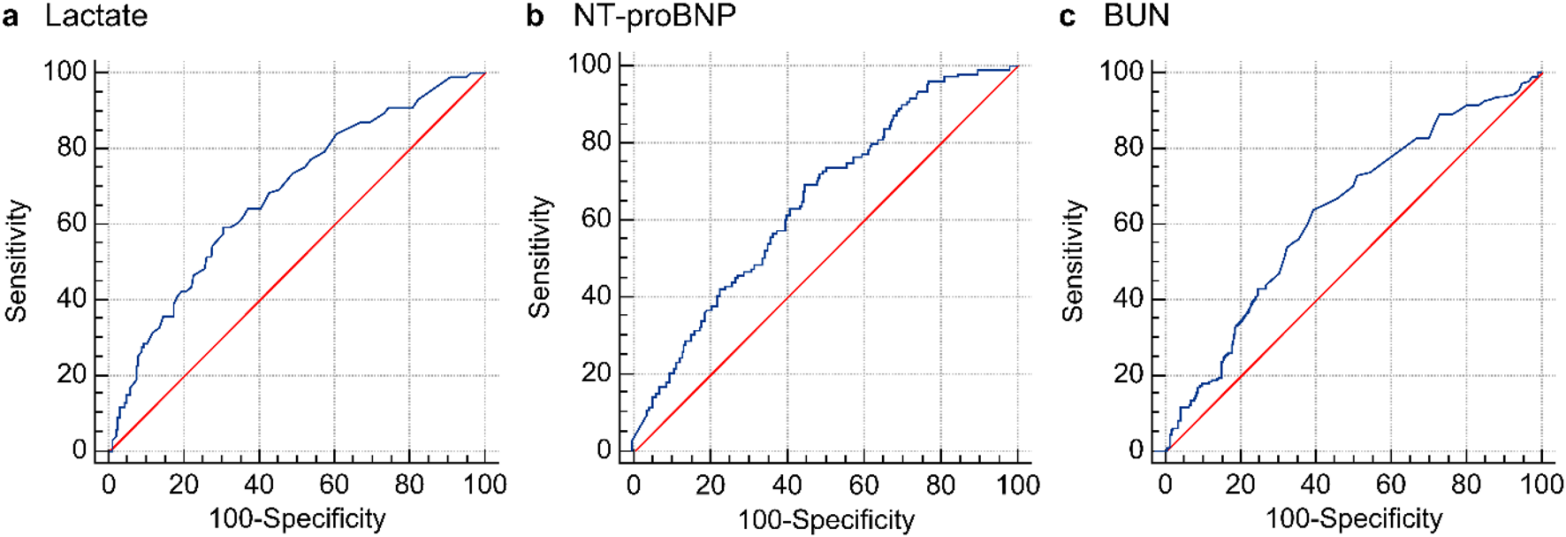
Receiver operating characteristic (ROC) curve analysis of lactate (**a**), NT-proBNP (N-terminal prohormone of brain natriuretic peptide, (**b**), and BUN (blood urea nitrogen, (**c**) of patients with septic shock for in-hospital mortality.

ROC curve analysis was performed for lactate, NT-proBNP, BUN, arterial pH, CRP, creatinine, and procalcitonin levels (*p* < 0.05; Table 3). The AUC values for predicting in-hospital mortality were as follows: Lactate, 0.678 (95% CI: 0.627–0.726); NT-proBNP, 0.648 (95% CI: 0.600–0.694); BUN, 0.630 (95% CI: 0.582–0.676), arterial pH, 0.618 (95% CI: 0.570–0.665); CRP, 0.607 (95% CI: 0.558–0.654); creatinine, 0.588 (95% CI: 0.539–0.636) and procalcitonin, 0.573 (95% CI: 0.522–0.624). ROC curves were compared using three blood tests with high AUROC values: lactate, NT-proBNP, and BUN (Fig. 2). In the comparisons between the two blood tests, lactate vs. NT-proBNP (*p* = 0.6278), lactate vs. BUN (*p* = 0.1667), and NT-proBNP vs. BUN (*p* = 0.3188), findings were not significantly different (Table 4).

**Table 3:** AUROC, cut-off value, sensitivity, and specificity for hospital mortality of patients with septic shock.

	*p*-value	Cutoff value	AUROC	(95% CI)	Sensitivity (%)	(95% CI)	Specificity (%)	(95% CI)	+ LR	(95% CI)	− LR	(95% CI)
Lactate (mg/dL)	< 0.0001	> 37	0.678	0.627–0.726	59.41	49.2–69.1	69.23	63.2–74.8	1.93	1.51–2.46	0.59	0.46–0.75
NT-proBNP (pg/mL)	< 0.0001	> 2591	0.648	0.600–0.694	69.37	59.9–77.8	54.72	49.0–60.4	1.53	1.29–1.82	0.56	0.42–0.75
BUN (mg/dL)	< 0.0001	> 34	0.630	0.582–0.676	63.96	54.3–72.9	60.46	54.7–66.0	1.62	1.33–1.97	0.60	0.46–0.78
pH, arterial	0.0002	≤ 7.322	0.618	0.570–0.665	41.44	32.2–51.2	78.62	73.6–83.1	1.94	1.42–2.64	0.74	0.63–0.88
CRP (mg/dL)	0.0008	> 10.7	0.607	0.558–0.654	69.37	59.9–77.8	48.85	43.1–54.6	1.36	1.15–1.60	0.63	0.46–0.85
Creatinine (mg/dL)	0.0063	> 1.51	0.588	0.539–0.636	65.77	56.2–74.5	50.65	44.9–56.4	1.33	1.12–1.59	0.68	0.51–0.89
Procalcitonin (ng/mL)	0.0249	> 5.57	0.573	0.522–0.624	53.33	43.3–63.1	59.64	53.6–65.5	1.32	1.05–1.66	0.78	0.62–0.98

*AUROC* area under the receiver operating characteristic curve; *CI* confidence interval; *LR* likelihood ratio; *NT-proBNP* N-terminal pro-brain natriuretic peptide; *BUN* blood urea nitrogen; *CRP* C-reactive protein.

**Table 4:** Pairwise comparison of the ROC curves.

		Difference between areas	(95% CI)	*p*-value
Lactate	NT-proBNP	0.0200	− 0.0608–0.101	0.6278
	BUN	0.0592	− 0.0247–0.143	0.1667
NT-proBNP	BUN	0.0392	− 0.0379–0.116	0.3188

## Discussion

In this study, we investigated the utility of NT-proBNP as a biomarker for predicting in-hospital mortality in patients presenting to the emergency department with septic shock. The results revealed that NT-proBNP was a valuable predictive factor comparable to lactate, emphasizing its significance in predicting in-hospital mortality.

NT-proBNP is widely used in the ED for the diagnosis of heart failure. NT-proBNP, a metabolite of pro-BNP, is a prohormone secreted by myocardial cells^24^. The prohormone of brain natriuretic peptide (proBNP) is decomposed into the active metabolite brain natriuretic peptide and the inactive metabolite NT-proBNP. NT-proBNP has a longer half-life, so it remains in the bloodstream for a longer period of time^25,26^. Natriuretic peptides are predominantly secreted during volume overload and cardiomyocyte stretching^27^. Although BNP is eliminated through various pathways, NT-proBNP is cleared exclusively by the kidneys^28–32^. Research comparing BNP and NT-proBNP is ongoing; however, there is still no consensus on which peptide is superior^33–37^, and the roles of these peptides have not been thoroughly studied^32,38^.

Although an increased plasma NT-proBNP level is primarily used as a biomarker for left ventricular (LV) dysfunction^26^, it is not necessarily specific to heart failure and is influenced by various conditions that cause myocardial strain and affect the clearance of NT-proBNP, including myocardial ischemia, arrhythmia, sepsis, shock, anemia, renal failure, pulmonary embolism, asthma, acute respiratory disease syndrome, and chronic obstructive pulmonary disease^32,36,39–42^. NT-proBNP levels can increase in various situations, particularly in septic shock, a systemic inflammatory response accompanied by multi-organ damage, and there may be diverse interpretations as to whether the elevated levels are indicative of LV dysfunction or other diseases^32,43–47^.

Since the underlying conditions can be difficult to ascertain in the ED, predicting the mortality rate in patients with septic shock using NT-proBNP levels, regardless of the underlying diseases, would be clinically useful. This analysis did not exclude patients with preexisting heart or kidney diseases. The survival and death groups included patients with various diseases, and the only significant difference between the two groups was stroke (*p* < 0.05, Table 1).

In this study, lactate had an AUC of 0.678 (95% CI: 0.627–0.726), and NT-proBNP had an AUC of 0.648 (95% CI: 0.600–0.694) as predictors of mortality in patients with septic shock, and this finding is consistent with previous research results^32,36,48–52^. There was no significant difference in the ROC curves between lactate and NT-proBNP (*p* = 0.6278, Table 4), indicating that NT-proBNP may serve as a substitute for lactate in predicting mortality in patients with septic shock when lactate cannot be used.

NT-proBNP could be a useful predictor of in-hospital mortality in patients with septic shock who present to the emergency department.

### Limitations

This study has several limitations. Owing to its single-center design, caution should be exercised when extrapolating and applying the research findings on a broader scale. Additionally, the retrospective nature of this study introduced inherent limitations, particularly in defining sepsis. In the ED, patients with suspected sepsis are routinely tested for their initial lactate levels. However, lactate levels are not monitored after adequate fluid resuscitation, which does not fulfill the definition of septic shock recommended by Sepsis-3^1^.

## Data Availability

The datasets used and analyzed during the current study are available from the corresponding author on reasonable request.
